# Guardians of precision: advancing radiation protection, safety, and quality systems in nuclear medicine

**DOI:** 10.1007/s00259-024-06633-w

**Published:** 2024-02-06

**Authors:** Francesco Giammarile, Peter Knoll, Jolanta Kunikowska, Diana Paez, Enrique Estrada Lobato, Miriam Mikhail-Lette, Richard Wahl, Ola Holmberg, May Abdel-Wahab, Andrew M. Scott, Roberto C. Delgado Bolton

**Affiliations:** 1 Department of Nuclear Science and Applications, Nuclear Medicine and Diagnostic Imaging Section, International Atomic Energy Agency, Vienna, Austria; 2 Nuclear Medicine Department, Medical University of Warsaw, Warsaw, Poland; 3 Washington University in St Louis School of Medicine, St. Louis, USA; 4 The Johns Hopkins University School of Medicine, Baltimore, USA; 5 Department of Nuclear Safety and Security, Radiation Safety and Monitoring Section, International Atomic Energy Agency, Vienna, Austria; 6 Department of Molecular Imaging and Therapy, Austin Health, Melbourne, Australia; 7 Olivia Newton-John Cancer Research Institute, Melbourne, Australia; 8 School of Cancer Medicine, La Trobe University, Melbourne, Australia; 9 Faculty of Medicine, University of Melbourne, Melbourne, Australia; 10 Department of Diagnostic Imaging (Radiology) and Nuclear Medicine, University Hospital San Pedro and Centre for Biomedical Research of La Rioja (CIBIR), La Rioja, Logroño, Spain; 11 Servicio Cántabro de Salud, Santander, Spain

**Keywords:** Nuclear medicine, Radiation protection, Theranostics guidelines, National and international regulations, Quality assurance

## Abstract

**Background:**

In the rapidly evolving field of nuclear medicine, the paramount importance of radiation protection, safety, and quality systems cannot be overstated. This document provides a comprehensive analysis of the intricate regulatory frameworks and guidelines, meticulously crafted and updated by national and international regulatory bodies to ensure the utmost safety and efficiency in the practice of nuclear medicine.

**Methods:**

We explore the dynamic nature of these regulations, emphasizing their adaptability in accommodating technological advancements and the integration of nuclear medicine with other medical and scientific disciplines.

**Results:**

Audits, both internal and external, are spotlighted for their pivotal role in assessing and ensuring compliance with established standards, promoting a culture of continuous improvement and excellence. We delve into the significant contributions of entities like the International Atomic Energy Agency (IAEA) and relevant professional societies in offering universally applicable guidelines that amalgamate the latest in scientific research, ethical considerations, and practical applicability.

**Conclusions:**

The document underscores the essence of international collaborations in pooling expertise, resources, and insights, fostering a global community of practice where knowledge and innovations are shared. Readers will gain an in-depth understanding of the practical applications, challenges, and opportunities presented by these regulatory frameworks and audit processes. The ultimate goal is to inspire and inform ongoing efforts to enhance safety, quality, and effectiveness in nuclear medicine globally.

## Introduction

In the intricate world of healthcare, nuclear medicine has carved a niche for itself, offering innovative solutions for both diagnostic and therapeutic purposes. To ensure the significant advancements and contributions of nuclear medicine to patient care, this document aims at offering an in-depth exploration into the multifaceted approaches to ensure that the highest safety and quality standards are maintained in this sensitive medical field.

The guidelines and regulations developed by both national and international regulatory bodies are assessed in depth. These are not static, but evolving sets of rules, responsive to the fast-paced technological innovations and advancements that characterize nuclear medicine. The document underscores the importance of adaptability in these regulations, ensuring that they are not only current but also comprehensive, covering the expansive scope of nuclear medicine and its intersections with other medical and scientific fields.

Audits are spotlighted for their instrumental role in upholding these standards. They reflect the current state of compliance with established standards and point toward areas that require enhancement. Through a systematic review process, audits reinforce a culture of excellence and continuous improvement, ensuring that the journey toward optimal safety and quality is ongoing and dynamic.

We aim to present the significant contributions of entities like the International Atomic Energy Agency (IAEA). A decade ago, the Agency published the general safety requirements in the document entitled “Radiation Protection and Safety of Radiation Sources: International Basic Safety Standards” (IAEA, 2014) that amalgamate the principles of scientific research, ethical considerations, and practical applicability [1]. These international standards are instrumental in ensuring a harmonized approach to safety and quality, promoting a universally high standard of practice across geographical and jurisdictional boundaries. The present manuscript also details the contributions of relevant professional societies such as the Society of Nuclear Medicine and Molecular Imaging (SNMMI) and the European Association of Nuclear Medicine (EANM).

Furthermore, the document highlights the integral role of international collaborations and partnerships. In a field as complex as nuclear medicine, the pooling of expertise, resources, and insights is not just beneficial but essential. It fosters an international community of practice where knowledge, innovations, and best practices are shared, ensuring that advancements in one corner of the globe can be rapidly adopted and adapted worldwide, elevating the global standard of nuclear medicine practice.

This comprehensive coverage aims not only to inform but also to inspire ongoing and future efforts to enhance safety, quality, and effectiveness in the practice of nuclear medicine.

## The facility and regulatory documents

Nuclear medicine is intricately woven into comprehensive regulatory frameworks. Both national and international regulatory bodies provide guidelines that champion safety, efficiency, and best practices. Whether focused on the construction and operation of facilities, the handling and disposal of radiopharmaceuticals, or the training and certification of professionals, they serve as the backbone of nuclear medicine practice.

The ever-evolving nature of these guidelines highlights the progressive essence of nuclear medicine. As technological advancements introduce new procedures and equipment, regulatory standards must adapt accordingly to ensure the utmost safety for both patients and medical practitioners. Furthermore, the practice of nuclear medicine does not exist in isolation; it often intersects with other medical fields and disciplines, necessitating a holistic approach to regulation.

Through collaborations with international professional organizations and intergovernmental agencies, a synchronized approach emerges that joins best practices from every corner of the globe. Such collaborations ensure that the same standards of care and safety are upheld worldwide, promoting the high standards of nuclear medicine practice.

Audits, both internal and external, play a pivotal role in this regulatory landscape. These systematic reviews assess the compliance of facilities with established standards, identifying areas of excellence and those requiring improvement. They reinforce the commitment to quality assurance, ensuring that facilities adhere to international standards.

In this light, the contributions of entities like IAEA are very important providing guiding beacons for professionals in the field, ensuring that practices align with the highest above mentioned standards [1].

In response to the evolving regulatory environment, common recommendations are needed to support healthcare professionals in understanding these requirements. As an example, the EANM, SNNMI, and IAEA have developed a guide on how to set up a theranostic center [2]. Despite the wide global variation in the regulatory, financial, and medical landscapes, this guide attempts to provide valuable information to enable interested stakeholders to safely initiate and operate theranostic centers. Despite regional differences in the specific regulation of radiation safety, it is crucial that professional societies continue to cooperate to develop such frameworks to help in launching new initiatives, complying with these requirements.

## The radiopharmaceuticals

The role of radiopharmaceuticals in nuclear medicine extends beyond their basic definition. These are compounds that represent the combination of chemistry, physics, and medicine and over the years have transformed the landscape of medical diagnostics and therapeutic realms.

The journey of radiopharmaceuticals begins in the research laboratories, where scientists research molecular structures and their interactions. As a result of years long rigorous research, selective compounds are being developed. Their ability to target specific molecular sites, from cancerous cells to specific regions of the brain, is a testimony to the precision and specificity that has been achieved in this field, ensuring minimal off-target effects and leading to more accurate diagnoses and targeted therapies.

The design of radiopharmaceuticals takes into account all desired patient- and disease-related effects. Their fast excretion rates ensure that following imaging or therapy the systemic exposure and potential side effects are minimized as much as possible. This rapid excretion, combined with the targeted nature of the compounds, underscores their safety profile and their role as invaluable tool in the arsenal of nuclear medicine practitioners.

As our understanding of human biology deepens and technology advances, it is expected that in the coming years the realm of radiopharmaceuticals is poised for more ground-breaking discoveries.

To allow in-house production of radiopharmaceuticals to be viable and to comply with an ever-changing regulatory environment, it is very important that radiopharmaceuticals are prepared in a standardized manner. Therefore, the EANM has issued recommendations, such as the “Guideline on current good radiopharmacy practice (cGRPP) for the small-scale preparation of radiopharmaceuticals” [3]. In addition, amid the reform of the European Pharmaceutical Legislation, the EANM has published various position papers and guidance documents on how the revised regulatory framework might impact the field of nuclear medicine [4, 5].

## The cameras and imaging systems

Modern advancements in imaging technology have dramatically reshaped the field of nuclear medicine. Devices such as those used for single photon emission computed tomography (SPECT) and positron emission tomography (PET) imaging have become fundamental pillars in the domain of nuclear medicine allowing medical professionals to visualize physiological and pathological processes at a cellular and molecular level.

The capabilities of these devices are not limited to providing images, they offer a window into the dynamic processes occurring within the human body. From tracking the spread of a cancerous tumor to monitoring the effectiveness of a therapeutic regimen, these devices provide clarity and precision with the dual purpose of disease diagnosis and treatment monitoring, often having a key role in patient management.

The efficacy of these systems is determined not only by the technology that is applied. It also depends on their service and maintenance. Periodic calibrations ensure the accuracy of the images, and routine maintenance checks provide the machines’ operational efficiency. Furthermore, periodic software updates help keep the systems updated with the latest advancements in imaging technology, ensuring that they remain at the forefront of diagnostic precision [6].

In the field of nuclear medicine, there is a pressing need for standardization [7]. Harmonization was the idea behind the creation of an accreditation program for PET scanners by the EANM. The EARL FDG-PET/CT accreditation program, which was launched in July 2010, helps imaging departments meet standard requirements, therefore improving comparability of data and ensuring essential quality assurance in daily clinical practice as well as high eligibility as a participant in multicenter trials.

International protocols, spearheaded by bodies like the IAEA, play a pivotal role in ensuring that equipment and methodologies are consistent and harmonized globally. This not provides the reliability of results across different centers which subsequently fortifies the trust in nuclear medicine’s diagnostic and therapeutic potential [8]. International guidelines and procedure standards produced by the main scientific associations are regularly updated and are key in the advancement in research and clinical applications in the field [7, 9–14]. Collaboration between all the stakeholders is necessary for integrating patient care, an example being the recommendations for multidisciplinary care of cancer patients, in which nuclear medicine has a relevant role [15–21]. In addition to the use of technologically advanced state-of-the-art equipment when available, attention to basic aspects such as the clinical history of the patient is fundamental for obtaining the maximum clinical benefit from the imaging procedure [22].

## The health personnel

Nuclear medicine services are provided by a diverse group of individuals, each bringing their own set of specialized skills to the table. Technologists with their in-depth knowledge of the technology ensure that the devices are operated optimally. More importantly, they come in close contact with the examined patients and are responsible for their comfort and lack of anxiety throughout the examination. Nuclear medicine physicians and radiologists, with their trained eyes, interpret the images, identifying abnormalities and providing important information regarding a patient’s health. Physicists ensure that the technology is used safely and efficiently and that correct doses of the radiopharmaceutical are administrated and perform, when needed, sophisticated quantitative measurements, such as dosimetry calculations, safeguarding the patients and the medical staff from potential radiation risks. The auxiliary, mostly administrative staff, often ensures the smooth functioning of the entire process.

The entire team is committed to continuous learning. The field of nuclear medicine is ever evolving, with new emerging developments and advancements. Periodical training sessions, workshops, and conferences are not just encouraged, they are imperative. These platforms ensure that professionals are updated with the latest in the field as well as equipped to integrate these advancements into their practice. Such rationale was key in establishing the European School of Multimodality Imaging & Therapy (ESMIT), the EANM educational response to huge challenges in the educational needs of the nuclear medicine community and the rising demand for more multimodality content.

Moreover, the essence of care in nuclear medicine transcends beyond diagnostics and treatment. It relies on compassion and dedication to patient well-being. The patient-centric approach these professionals adopt ensures that every patient is treated as an individual, with management strategies tailored to the unique needs, making the experience holistic and comprehensive.

In essence, while technology and machines are indispensable to nuclear medicine, it is the people behind them—their expertise, dedication, and compassion—that truly drive the field forward [23].

## The radiopharmaceutical administration protocols

The process of administering radiopharmaceuticals in nuclear medicine has to adhere to precise scientific protocols for every step, from the dosage calculation to the administration. An accurate dose can be the difference between an effective treatment and potential complications.

Firstly, radiopharmaceuticals are not just ordinary medicines. They are specially designed compounds labeled with radioactive isotopes tailored to target specific sites within the body. The preparation phase involves meticulous measurements and calibrations, ensuring that the right amount of radioactivity is the dose to be delivered.

Once prepared, the administration process is governed by well-established protocols. The first step is verifying the patient’s identity, ensuring that the right patient receives the right dose. This is followed by the actual injection, carried out by trained professionals who monitor the patient for any immediate adverse reactions. The fact that these compounds are radioactive makes the monitoring phase even more crucial.

As advancements are made, the protocols undergo revisions based on feedback from professionals and ongoing research, being updated and optimized for patient safety and efficacy.

These requirements are underscored by international guidelines and standards, such as those set by the IAEA, EANM, and SNNMI [7, 9–21]. These standards serve as a benchmark, ensuring that practices worldwide adhere to a gold standard of safety and quality.

The administration of radiopharmaceuticals has to be performed routinely based on complex protocols and guidelines prioritizing patient safety above all [24].

## The computer processing, advanced analysis, and artificial intelligence

In an era where technology rapidly permeates every facet of our lives, the medical sector stands as one of the most profound beneficiaries. Nuclear medicine, a discipline at the forefront of innovative health solutions, has broadened its capabilities by embracing the vast potential of digital advancements, particularly in the realm of artificial intelligence (AI) and machine learning (ML). An example of how technological advancement allow more information to be extracted from medical images is radiomics, also denominated image mining. It consists in analyzing aspects of the image not visible to the human eye, which can provide information of the characteristics of the tissues. Research is being carried out in search of radiomic features that may be used as imaging biomarkers [25] and specifically for those that include a combination of imaging and clinical features, ranging from the use of different radiotracers to the incorporation of various clinical-radiological features [26, 27].

ML, a subset of AI, identifies intricate patterns within large datasets, often capturing nuances that human experts might overlook. When applied to nuclear medicine, this computational prowess can lead to enhanced diagnostic accuracy, faster processing times, and identify previously unrecognized patterns in imaging data. Deep learning, a more specialized form of ML, uses neural networks to process information in ways inspired by the human brain leading to significant enhancements in imaging techniques.

Beyond just diagnostics, AI’s foray into nuclear medicine has transformative implications for treatment planning and patient management. Algorithms can now predict patient responses to specific treatments, optimize dosing regimens based on individual patient profiles, and even assist in real-time decision-making during procedures. These AI-driven insights can lead to more personalized treatment plans, ensuring optimal outcomes for each patient.

Moreover, as we continue to generate vast amounts of medical data every day, the importance of effective and efficient data processing becomes even more pronounced. With AI and advanced computational methods, high-speed large-scale data analysis can be performed in order to extract significant meaningful information. However,

as with all technological advancements, there is a need for caution. The integration of AI into nuclear medicine must be accompanied by rigorous validation and testing, ensuring that these algorithms are reliable and robust. Collaborative efforts between AI experts, nuclear medicine physicians and radiologists, are crucial to harness the full potential of this synergy while maintaining the highest standards of patient care.

As we stand at the intersection of nuclear medicine with AI and advanced computer processing, it is to be predicted that they will allow for future breakthroughs, reshaping nuclear medicine and setting new standards for patient care and treatment outcomes.

## Dosimetry: challenges and modern solutions

Dosimetry, the science of determining the amount and distribution of radiation dose absorbed by the human body, is a cornerstone of nuclear medicine and radiation therapies. It is highly important, especially when the margin between therapeutic effect and potential harm can be razor-thin. While delivering the precise dose is integral to patient safety, it also directly impacts the efficacy of the therapy [28].

Several state-of-the-art software tools and technological innovations have considerably advanced the precision of dosimetry. These tools can model radiation within the body, calculate dose distributions, and even predict potential side effects based on the absorbed dose. As powerful as these tools are, they are continuously being refined and enhanced to provide even more accurate and patient-specific dosimetry data [29].

Like all evolving fields, dosimetry faces its own set of challenges [30]. One of the primary issues is the integration and comparison of data across different dosimetry systems and methodologies. Each system may have its own set of assumptions, algorithms, and calibration standards. This diversity can pose hurdles when attempting to standardize practices or compare outcomes from different institutions or studies. There is also the challenge of keeping up with the rapidly advancing technology in imaging and therapy, as these advancements often necessitate updates and recalibrations in dosimetry tools.

Moreover, there is an increasing need to develop real-time dosimetry solutions, allowing practitioners to adjust treatment parameters on-the-fly based on immediate feedback. Such capabilities can further enhance the safety and effectiveness of treatments, ensuring that patients receive the optimal dose while minimizing potential side effects.

Solutions to these challenges are on the horizon. Collaboration between researchers, clinicians, and industry is fostering the development of standardized protocols and methodologies. Newer algorithms, leveraging the power of AI and ML, are being developed to process and analyze dosimetry data efficiently and accurately. There is also growing emphasis on training and education, ensuring that professionals in the field are well-equipped to utilize these advanced tools and navigate the complexities of modern dosimetry.

The overreaching goal remains unchanged, to harness the therapeutic potential of radiation while ensuring utmost patient safety as we continue to push the boundaries of what is possible in radiation therapies. It is a challenge the field is well poised to meet, backed by relentless research, technological advancements, and a commitment to excellence [28, 29].

## The ongoing image interpretation quality, modern techniques, and continuous improvement

Image interpretation in nuclear medicine is a blend of art and science. It calls for an understanding of both the technology behind the images and the human anatomy and physiology they depict. Historically, this field relied heavily on the expertise and experience of radiologists and nuclear medicine specialists to decipher these images and derive meaningful clinical insights [31].

However, nuclear medicine is rapidly changing. With the advent of AI and ML, the capabilities of image interpretation have been significantly enhanced. When paired with advanced imaging techniques, those tools offer a much more in-depth view of physiological and pathological processes. AI algorithms, especially those harnessing the power of DL, can analyze vast datasets and highlight subtle patterns or abnormalities [25, 32, 33].

However, this does not mean that human expertise is becoming obsolete—quite the contrary. The fusion of human expertise with AI augments the overall quality of image interpretation. Professionals in the field can leverage AI to sift through the data, but the final judgment, especially in complex cases, often rests upon the experts. This synergy ensures a more accurate, efficient, and comprehensive analysis.

The global nuclear medicine community recognizes the importance of maintaining the highest standards of image interpretation. Regular peer reviews are now a staple in many institutions, sharing insights and feedback. International workshops, conferences, and training programs serve as platforms for knowledge exchange to learn about the latest advancements, share research findings, and discuss challenging cases, fostering a culture of continuous learning.

Training curricula are evolving, traditional programs being now supplemented with modules on AI, ML, and other emerging topics. This will ensure that the upcoming generation of nuclear medicine professionals is well equipped to harness the full potential of these tools.

In essence, the realm of image interpretation in nuclear medicine is undergoing a transformative phase. While the tools and techniques are evolving, the core objective remains unchanged: to provide the highest quality of patient care [25, 32, 33].

## Therapy protocols in nuclear medicine and their evolution

Nuclear medicine is at the forefront of innovative therapeutic solutions. Its applications span a range of medical conditions, in patients who previously had limited treatment options, mainly involved with targeted treatment of various malignancies using radioisotopes and palliative management of chronic pain [9, 13, 34–36].

It is possible to target malignant cells at the molecular level, sparing the surrounding healthy tissues. This specificity reduces side effects and enhances the therapeutic efficacy of the treatment delivering a therapeutic dose directly to the malignancy [35, 37].

The development of these intricate therapy protocols is no small feat. It requires rigorous research and clinical trials to ensure both safety and effectiveness, each of them being the result of countless hours of research, experimentation, and validation.

New discoveries in molecular biology, advancements in radiopharmaceuticals, and innovations in imaging technologies all contribute to this ever-evolving field. For instance, the development of newer radionuclides with optimal decay properties allows for more effective and safer treatments. As the understanding of these areas deepens, treatment options and protocols become more refined and targeted [35]. 

Moreover, the collective expertise and collaboration between nuclear medicine professionals, oncologists, radiopharmacists, and molecular biologists foster a multidisciplinary cutting-edge and holistic approach to treatment [15–21].

With the continued push for research and the sharing of knowledge across the globe, the future of therapeutic applications in nuclear medicine is promising. It holds the potential to change the trajectory of many diseases, offering patients not just treatment but a chance for a better quality of life.

The global community, including organizations such as the IAEA, EANM, and SNMMI, plays a crucial role in standardizing and disseminating these protocols. Their guidelines and preprints ensure the uniformity in treatment approaches, ensuring that patients, irrespective of their geographical location, have access to the best possible care [7, 9–21, 34].

The growing trajectory of nuclear medicine is evident from the increasing trends in its applications, especially in countries with advanced healthcare infrastructure. As more facilities adopt and integrate nuclear medicine into their therapeutic arsenal, it is poised to become a mainstay in modern medicine [34–37].

## Accreditation, global perspectives, and the path forward

Accreditation in the practice of nuclear medicine is a testimony to the quality and excellence of a facility, a sign of trust and assurance for both patients and medical professionals. When a renowned professional institution accredits a facility, it sends a strong message about its commitment to uphold the highest standards of safety, expertise, and patient care. This rigorous process of accreditation involves comprehensive evaluations, regular audits, and adherence to international benchmarks [6].

In today’s globalized world, the practice of medicine, mainly in specialized fields like nuclear medicine, does not exist in isolation. It is a collaborative effort that transcends borders. The rapid dissemination of research findings, innovative methodologies, and cutting-edge technologies is made possible through international collaborations and partnerships [7, 9–21, 34]. These global exchanges are pivotal in advancing the field, ensuring that innovations benefit patients worldwide.

There is also a notable emphasis on standardization in nuclear medicine. Given the intricate nature of the procedures and the potential risks associated with radiation, it is imperative that there is uniformity in practice. This standardization ensures that regardless of where a patient receives treatment—be it in North America, Europe, Asia, or any other part of the world—the quality of care remains consistent [7].

Another significant aspect of the global perspective is the emphasis on equality in training and continuous education. With the field of nuclear medicine evolving at a breakneck pace, it is crucial for professionals to stay updated with the latest advancements and international meetings and training programs provide platforms for knowledge exchange, fostering continuous professional improvement.

The path forward for nuclear medicine is promising. With the convergence of technology, biology, and clinical practice, the possibilities are endless. From early and precise diagnosis to targeted therapies, nuclear medicine is poised to redefine the paradigms of healthcare. But the journey ahead also necessitates a collective effort. It calls for collaboration, a commitment to excellence, and an unwavering focus on patient-centric care.

While accreditation is a significant milestone, it is just one of the many steps in the journey toward excellence. The global nuclear medicine community must continue to collaborate, innovate, and strive for perfection.

## Conclusion

As we navigate the complexities of the modern healthcare domain, nuclear medicine emerges as a beacon of transformative potential. Intertwining advanced technology, ground-breaking research, and compassionate patient care, it represents a paradigm shift in how we approach diagnostics and therapeutics. Its capabilities, from precise targeting of diseases at the molecular level to offering innovative therapeutic solutions, are truly revolutionary. In the face of evolving healthcare challenges, nuclear medicine is bridging gaps between what we know and what we aspire to achieve. With a staunch commitment to safety and highest quality standards and an insatiable drive for innovation, the future of nuclear medicine is not just promising—it is poised to set new benchmarks in global healthcare. As we look ahead, it is evident that the best chapters of nuclear medicine’s story are yet to be written, and its full potential is yet to be realized.
